# The role of self-math overlap in understanding math anxiety and the relation between math anxiety and performance

**DOI:** 10.3389/fpsyg.2015.01543

**Published:** 2015-10-14

**Authors:** Elizabeth A. Necka, H. Moriah Sokolowski, Ian M. Lyons

**Affiliations:** ^1^Department of Psychology, University of ChicagoChicago, IL, USA; ^2^Department of Psychology, University of Western OntarioLondon, ON, Canada

**Keywords:** math anxiety, math ability, math performance, self-math overlap, inclusion of other in self

## Abstract

Recent work has demonstrated that math anxiety is more than just the product of poor math skills. Psychosocial factors may play a key role in understanding what it means to be math anxious, and hence may aid in attempts to sever the link between math anxiety and poor math performance. One such factor may be the extent to which individuals integrate math into their sense of self. We adapted a well-established measure of this degree of integration (i.e., self-other overlap) to assess individuals’ self-*math* overlap. This non-verbal single-item measure showed that identifying oneself with math (having higher self-math overlap) was strongly associated with lower math anxiety (*r* = -0.610). We also expected that having higher self-math overlap would leave one especially susceptible to the threat of poor math performance to the self. We identified two competing hypotheses regarding how this plays out in terms of math anxiety. Those higher in self-math overlap might be more likely to worry about poor math performance, exacerbating the negative relation between math anxiety and math ability. Alternatively, those higher in self-math overlap might exhibit self-serving biases regarding their math ability, which would instead predict a decoupling of the relation between their perceived and actual math ability, and in turn the relation between their math ability and math anxiety. Results clearly favored the latter hypothesis: those higher in self-math overlap exhibited almost no relation between math anxiety and math ability, whereas those lower in self-math overlap showed a strong negative relation between math anxiety and math ability. This was partially explained by greater self-serving biases among those higher in self-math overlap. In sum, these results reveal that the degree to which one integrates math into one’s self – self-math overlap – may provide insight into how the pernicious negative relation between math anxiety and math ability may be ameliorated.

## Introduction

Research on interpersonal relationships suggests that as close relationships develop, each member of the relationship begins to incorporate the other member into his or her sense of self, fostering a sense of ‘self-other overlap’ and leading to greater valuation of and commitment to their partner and the relationship (Aron et al., 1992; Agnew et al., 1998; Aron and Fraley, 1999). Self-other overlap was originally conceptualized as a measure of interpersonal closeness between two members of a relationship. However, recent research has demonstrated that non-human and abstract entities, such as sports (Blanchard et al., 1998), nature (Schultz, 2001), consumer brands (Reimann et al., 2012; Trump and Brucks, 2012), and God (Hodges et al., 2013) can also be incorporated into one’s sense of self in a manner similar to integrating another person into one’s self, and can produce comparable effects. For example, individuals with high self-brand overlap are more likely to confuse attributes associated with a favorite brand with attributes associated with the self (Trump and Brucks, 2012), and individuals with high self-nature overlap are more likely to engage in behavior that benefits nature (Davis et al., 2009).

It is possible that some people highly value mathematics or view their interest and success in math as an integral part of who they are. In a cyclical process, integration of math into their sense of self may foster even greater valuation of and engagement in mathematics, akin to the way in which including a close relationship partner in the self enhances relationship development. Indeed, research examining the concept of math identification suggests that one’s level of math identification (the degree to which individuals perceive math as self-relevant and important) can predict motivation to study for math exams (Smith and White, 2001) and greater likelihood of considering STEM careers (Smith et al., 2005). If individuals identify strongly with math, then their success in math should be a highly valued goal for which they are “self-evaluatively accountable” (Steele, 1997, p. 613), and failure in that domain should have important negative implications for one’s sense of self-worth. Thus, in much the same way that including a close relationship partner in one’s sense of self modifies an individual’s perceptions of and behaviors toward the relationship, integrating math into one’s self may produce distinct psychological and behavioral consequences for one’s relationship with math. That said, most measures of math identification rely heavily on assessments of one’s own math ability^1^, whereas work from the self-other overlap literature indicates that various additional factors (e.g., perceptions of another’s inclusion of you in their self, frequency of time spent together, etc.; Aron et al., 1992) contribute to the strength of a given self-other overlap rating. Because self-math overlap makes no presuppositions about perceptions of math achievement, it permits us to capture additional variance that might not be captured by self-report measures of math identification (e.g., those for whom math is particularly valued and personally relevant but who perceive that their math abilities are not especially strong).

**FIGURE 1:**
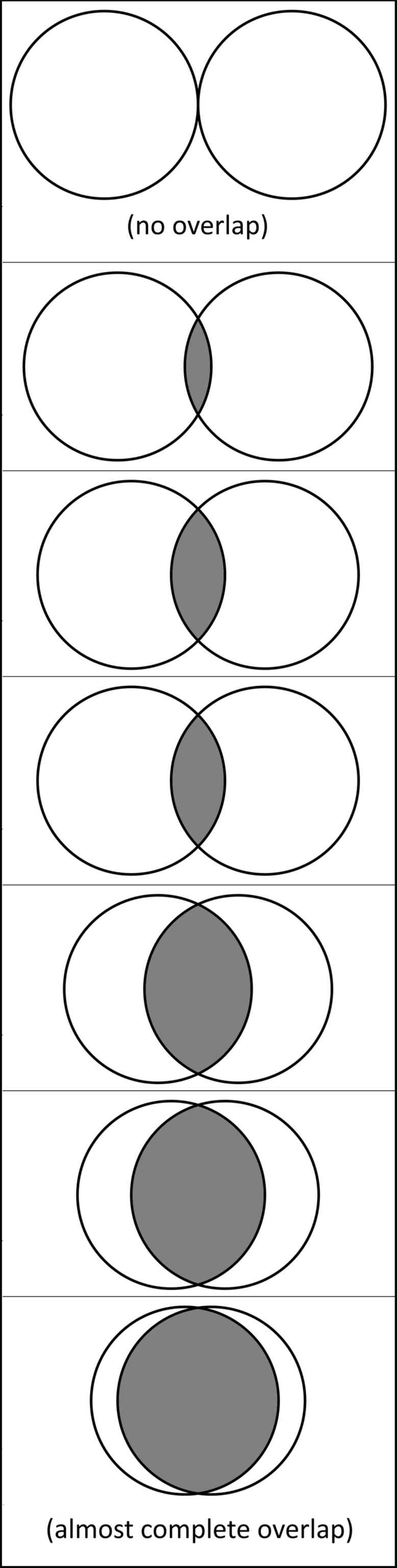
**The Self-Math Overlap measure**. Participants selected the item which best represented their relationship with mathematics, where one circle represented their self (i.e., “You”) and the other circle represented mathematics.

Of particular interest in the current study is how one’s inclusion of math in self relates to one’s feelings of math anxiety. It is becoming increasingly evident that psychosocial factors play a key role in the experience of math anxiety (Meece et al., 1990; Pajares and Miller, 1994; Pekrun, 2006; Beilock et al., 2010; Ahmed et al., 2012). Furthermore, people who value math tend to experience less math anxiety (Hembree, 1990; Meece et al., 1990), and implicit measures of math identification are associated with implicit anxiety toward math (Nosek and Smyth, 2011). Here, we adapted a widely used measure of self-expansion in close relationships, the Inclusion of Other in Self Scale (IOS; Aron et al., 1992), to assess the extent to which an individual’s cognitive representations of math and self are overlapping (a measure we call ‘self-math overlap’; see **Figure 1**). This simple measure takes less than a minute to complete, and its visual nature potentially lends itself to use in a wide range of settings and participants. Drawing from theories of interpersonal relationship development, domain identification, motivation, and math anxiety, we hypothesized that individuals who integrate math into their sense of self (e.g., have higher self-math overlap) would value math more and would also report lower levels of math anxiety using a traditional and widely used math anxiety scale (sMARS; Alexander and Martray, 1989).

However, in addition to these psychosocial factors, cognitive factors also contribute to math anxiety. It is well known, for instance, that there exists a persistent negative relation between poor math skills (a cognitive factor) and high math anxiety (for a review, see Ashcraft and Ridley, 2005). Furthermore, one’s math achievement, one’s beliefs about one’s math abilities, and genetic factors associated with math problem-solving skills are predictive of math anxiety (Ma and Xu, 2004; Goetz et al., 2013; Wang et al., 2014). Understanding the psychosocial factors that modulate the strength of the association between math performance and math anxiety and how they do so is critical for decoupling this pernicious negative cycle. An individual’s degree of self-math overlap may be one such factor. If math is an important part of the self (i.e., self-math overlap is higher), then being ‘good’ at math should be important for maintaining self-integrity, or the belief that the self is good, virtuous, and able to control important life outcomes (Steele, 1988; Sherman and Cohen, 2006). An individual who values but is unable to perform well in math may develop negative self-evaluations and view himself as inadequate, incapable, or otherwise flawed. Conversely, an individual for whom math is not integrated into self should exhibit fewer self-evaluative concerns while doing math and thus poor math performance should minimally threaten perceptions of self-integrity. Yet the way that individuals potentially deal with the threat to self-integrity (or relative lack thereof) of being ‘bad’ at math is unclear.

One possibility is that those higher in self-math overlap succumb to the threat to their sense of self of poor math performance^2^, such that the negative relation for these individuals between math performance and math anxiety might be exacerbated. From this point of view, threat of math failure among individuals with higher levels of self-math overlap (and the ensuing damage that such math failure might do to their perceptions of self) might be especially acute. Indeed, evidence suggests that in the presence of negative stereotypes about one’s math abilities (i.e., stereotype threat), women who strongly identify with math exhibit impaired math performance (Steinberg et al., 2012). Both stereotype threat and math anxiety are thought to predict poor math performance in part because the worries and distraction related to the experience of the threat or anxiety consume valuable cognitive resources that are necessary to successfully complete the task (Hopko et al., 1998; Schmader and Johns, 2003; Ashcraft and Krause, 2007; Beilock and Gray, 2007; Beilock et al., 2007). This distraction or worry appears to be exacerbated – at least in the case of stereotype threat – by strongly identifying with the domain in which one’s performance is negatively evaluated (Steinberg et al., 2012). In a similar vein, it is possible that individuals with higher self-math overlap would also be most susceptible to the cognitively depleting effects of math anxiety. From this perspective, individuals higher in self-math overlap should demonstrate an exacerbated negative relation between poor math performance and math anxiety. Taking this view further, we would expect that for individuals with relatively low self-math overlap, the possibility of math failure should be minimally threatening to one’s sense of self. Because these individuals’ math ability is not meaningfully contributing to their self-integrity, math performance outcomes are less important to them and math should be a much less worry-inducing task. This in turn would potentially diminish the negative loop between poor math performance and math anxiety. In other words, individuals who integrate math into the self less (i.e., have lower self-math overlap) should demonstrate a decoupled relation between math anxiety and math performance (i.e., a reduced or even eliminated negative relation).

An alternative perspective is that the threat of math failure may promote a defensive response among those higher in self-math overlap, such that they employ protective cognitive biases to ameliorate the perceived threat (Gilbert et al., 1998; Sherman and Cohen, 2006). Individuals are motivated to arrive at conclusions which place the self in a favorable light (Kunda, 1987, 1990; Taylor and Brown, 1988). When individuals are motivated to maintain high levels of self-regard in a particular domain or area (e.g., math), one way that they may do so is through self-serving biases (Dunning et al., 1995). Thus, to reduce the threat of math failure (and the ensuing damage this might do to their perception of self), people higher in self-math overlap might overestimate how good they are at math, such that there is discordance between their perceived and objective math ability. Such overly positive expectations of their math performance could serve to insulate these individuals from the deleterious effects of ruminating about potentially poor math performance (Hopko et al., 1998; Pekrun, 2006; Ashcraft and Krause, 2007; Beilock and Gray, 2007). From this perspective, math failure should not be as threatening to self-integrity among those with lower self-math overlap and so would not be expected to promote a defensive response in them. Thus, these self-serving biases might be absent for individuals with lower self-math overlap, and their more precise perceptions of their math abilities (and any potential deficiencies therein) would in turn predict a stronger negative relation between math anxiety and math performance for those on the lower end of the self-math overlap spectrum compared to those on the higher end. In sum, this second hypothesis predicts that the more that math is integrated into the self (i.e., the higher one’s self-math overlap), the more we should see a decoupling of the negative relation between math anxiety and math performance, a decoupling which may be explained – at least in part – by increased self-serving biases.

To summarize, in the present study we examined whether self-math overlap relates to math anxiety and the extent to which individuals value math. Furthermore, we tested two competing hypotheses (outlined above) regarding whether one’s degree of self-math overlap moderates the relation between math performance and math anxiety. We also assessed the extent to which math self-serving bias may or may not explain (i.e., mediate) the potential moderating effect of self-math overlap.

## Materials and Methods

### Participants

First-year University of Western Ontario undergraduate students were recruited as part of a larger study examining academic decisions in undergraduates. Participants were recruited through flyers which were placed on public bulletin boards randomly throughout campus and through online advertisements on Facebook and other social networking groups for first-year University of Western Ontario students. Recruitment materials made no mention of mathematics. From an initial sample of 186, two participants were excluded because they were not actually first year students and three participants were excluded for failing to meet *a priori* exclusion criteria (i.e., incorrectly answering more than one third of instructional manipulation check items; Oppenheimer et al., 2009), resulting in a total of 181 participants (66 males, 115 females, aged 17–20, *M* = 18.55, *SD* = 0.39).

### Procedure

Data reported here are part of a larger dataset focusing on first-year undergraduates. All present measures were obtained in a single 2 h session in which participants completed a series of cognitive tasks and self-report measures. The order of the tasks was counterbalanced across participants, and the order of the questionnaires within the survey battery was randomized across participants. All cognitive tasks were presented using EPrime 2.0 and all surveys were presented through Qualtrics (Provo, UT, USA). Participants were seated at identical Dell desktop machines running Windows 8.1 roughly 60–70 cm from the screen (flat-screen LCD monitor). Participants completed the math and verbal task via keyboard input and all surveys and other tasks via mouse input. The session took approximately 2 h to complete, and all participants were compensated $20 CAD. All participants provided written consent and all procedures were approved by the University of Western Ontario Ethics Review Board.

### Materials

All summary statistics of survey and behavioral measures are presented in **Table 1**.

**Table 1:** Descriptive statistics and correlation matrix of survey and behavioral measures.

	Measure	*M (SD)*	1	2	3	4	5	6	7	8	9	10	11	12
1	Self-math overlap	3.24 (1.63)												
2	Math anxiety	30.62 (20.65)	-0.61^∗∗∗^											
3	Math performance	50.64 (24.63)	0.35^∗∗∗^	-0.36^∗∗∗^										
4	Valuation of math	10.27 (5.04)	0.73^∗∗∗^	-0.66^∗∗∗^	0.32^∗∗∗^									
5	Perceived math ability	1.89 (1.06)	0.70^∗∗∗^	-0.71^∗∗∗^	0.37^∗∗∗^	0.75^∗∗∗^								
6	Math bias	-	0.61^∗∗∗^	-0.62^∗∗∗^	-	0.68^∗∗∗^	0.93^∗∗∗^							
7	Trait anxiety	40.94 (10.73)	-0.21^∗∗^	0.44^∗∗∗^	0.02	-0.23^∗∗^	-0.30^∗∗∗^	-0.33^∗∗∗^						
8	Working memory capacity	40.06 (15.57)	0.03	-0.06	0.13†	0.02	-0.02	-0.08	-0.08					
9	Self-literature overlap	2.91 (1.52)	-0.23^∗^	0.18^∗^	-0.17^∗^	-0.18^∗^	-0.15^∗^	-0.10	0.07	0.12				
10	Self-friend overlap	4.79 (1.35)	-0.03	0.17^∗^	0.00	-0.11	-0.11	-0.11	0.01	-0.01	0.07			
11	Verbal performance	-0.01 (0.75)	0.06	-0.22^∗∗^	0.13†	0.15^∗^	0.19^∗^	0.15^∗^	-0.07	0.15^∗^	0.23^∗∗^	-0.07		
12	Perceived reading ability	2.24 (0.93)	-0.06	-0.13†	-0.16^∗^	-0.03	0.02	0.08	-0.23^∗∗^	0.17^∗^	0.46^∗∗∗^	-0.08	0.37^∗∗∗^	
13	Literature bias	-	-0.09	-0.05	-0.23^∗∗^	-0.09	-0.06	0.03	-0.22^∗∗^	0.12†	0.40^∗∗∗^	-0.06	-	0.93^∗∗∗^

*Covariates are shown in grey. *N* = 181. †*p* < 0.10, ^∗^*p* < 0.05, ^∗∗^*p* < 0.01, ^∗∗∗^*p* < 0.001. *M*(*SD*) for bias measures are by definition 0(1) – see Methods for details. Note also that bias scores are residualized against math and verbal performance, respectively, so means for bias scores and correlations between a bias and a performance score are necessarily 0*.

#### Math Anxiety

Participants completed the short math-anxiety rating scale (sMARS; Alexander and Martray, 1989), in which they rated how anxious they feel in 25 math-related situations, such as “receiving a math textbook” and “walking to math class.” Items were scored on a 0–4 scale, with a higher value indicating higher anxiety, and summed for a composite measure of 0–100, with a higher value indicating higher math anxiety (Cronbach’s α = 0.96).

#### Self-Math Overlap

Participants completed a modified version of the Aron et al. (1992) IOS scale. Participants saw a series of seven Venn-diagrams with varying degrees of overlap, ranging from no overlap to almost complete overlap, and were instructed to indicate “how much your sense of yourself overlaps with the [specified] person or concept” (see **Figure 1**). To assess the unique contribution of including math in the self, relative to having a more complex self-concept, participants completed the IOS regarding their relationship with math as well as with their best friend and with literature. This resulted in three unique measures: self-math overlap, self-friend overlap, and self-literature overlap. Items were scored on a 0–6 scale, with a higher value indicating higher overlap.

#### Valuation of Math

Participants reported the extent to which they agreed with a number of statements regarding their views on mathematics. Statements were derived from the two motivation measures included in PISA 2012 (OECD, 2012): the intrinsic motivation to learn mathematics scale (INTMAT) and the instrumental motivation to learn mathematics scale (INSTMOT), a measure of extrinsic motivation. Two items were excluded from the INSTMOT scale to reduce the length of the survey. Example items include, “I look forward to my mathematics” (INTMAT), “I am interested in the things I learn in mathematics” (INTMAT), and “Mathematics is an important subject for me because I need it for what I want to study later on” (INSTMOT). The six items were scored on a 0–3 scale, with higher scores indicating greater agreement. Scores from the two scales were analyzed independently and were also summed to compute a composite measure of valuation of math (range: 0–18, with higher scores indicating greater valuation of math; Cronbach’s α = 0.91).

#### Trait Anxiety

Participants completed the 20-item trait anxiety inventory (TAI; Spielberger et al., 1970), which assesses how frequently participants experience generalized feelings of anxiety and calmness. The TAI was included to partial out any variance in math anxiety that is not specific to anxiety about math but rather is driven by overall anxiety. Items were scored on a 1–4 scale, with a higher value indicating higher anxiety, and were reverse coded where appropriate. Scores were summed for a composite measure of 20–80, with a higher value indicating higher trait anxiety (Cronbach’s α = 0.93).

#### Math Performance

Participants completed mental arithmetic problems and reported solutions in a free-response manner. Task trials were designed to be challenging and were adapted from the Kit of Factor-Referenced Cognitive Tests (Ekstrom et al., 1976; see also Lyons and Beilock, 2011). Trials were of four different operation types (addition, subtraction, multiplication, and division), and operations were presented in separate blocks, which were randomized across participants. (Examples problems: *Addition:* 49 + 27 + 36, 66 + 89 + 32; *Subtraction:* 551 - 268, 461 - 157; *Multiplication:* 71 × 9, 97 × 4; *Division:* 711 ÷ 3, 568 ÷ 8; note that all problems were presented vertically.) Each block lasted approximately 3 min, or until the participant completed their last trial if they were mid-trial when the 3 min elapsed. Importantly, participants were unaware of this time limit, thus alleviating the task of overt time-pressure. Math performance was measured as the total number of correctly solved problems within 3 min and was summed across all four blocks (higher score corresponds to higher math ability).

#### Verbal Performance

Participants completed a synonym matching task in which they were presented with a target word and proceeded to determine which of five words was most synonymous with the target word. Responses were made in a multiple-choice format. (Example items: *Target word:* Replete; *Potential synonyms:* Full, Elderly, Resentful, Discredited, Restful.) Trials were adapted from the Kit of Factor-Referenced Cognitive Tests (Ekstrom et al., 1976) and were designed to be somewhat challenging. Each trial lasted a maximum of 15 s. Participants completed one block of five practice trials, followed by two blocks of 18 trials each, summing to a total task time of about 5–6 min. Performance is measured via a combination of response-times and error-rates (*z*-scores for each measure were computed and averaged). Verbal performance was included to compute individuals’ potential self-serving biases with respect to performance in a domain outside of math. As noted above, inclusion of such control measures allows us to ascertain the extent to which any bias effects observed are specific to math.

#### Perceived Math Ability and Math Bias

Participants reported their perceived math ability by responding to the single item, “I am just not good at math,” adapted from the PISA index of mathematics self-concept (SCMAT; OECD, 2012). The item was reverse coded and scored on a 0–3 scale, such that higher scores indicate greater perceived ability. Previous work has demonstrated that perceived math ability is an important predictor of math anxiety (Ahmed et al., 2012) and is a path through which math anxiety exerts an effect on math performance (Meece et al., 1990). Here, we used this measure to compute individuals’ potential self-serving biases with respect to math performance.

To compute a measure of self-serving math bias, we entered math performance (as measured by scores on the mental arithmetic task) as a predictor into a linear regression model predicting perceived math ability. We reasoned that any variance in perceived math ability that cannot be attributed to differences in individuals’ math performance (a measure of their objective math ability) – i.e., perceived math ability residualized via the removal of the influence of actual math ability – would reflect a bias in one’s assessment of one’s math ability. Put another way, our math bias score is equivalent to perceptions of math ability that are independent of (i.e., orthogonalized with respect to) objective math performance. Positive bias scores thus indicate the presence of self-enhancing perceptions (i.e., an overestimation of one’s ability, relative to the rest of the sample), whereas negative bias scores indicate self-deprecatory perceptions (i.e., an underestimation of one’s ability, relative to the rest of the sample). Scores ranged from -2.44 to 1.64.

To assess biases specific to math rather than to general abilities, participants also completed an item assessing perceived literature ability, “I am just not good at reading,” which was also reverse coded and scored on a 0–3 scale. To ensure that bias was specific to math, we computed a measure of literature bias in a similar fashion to use as a covariate in subsequent analyses (perceived literature bias ratings were orthogonalized with respect to verbal performance; range = -3.06 to 1.83).

#### Working Memory

As a measure of working memory capacity, participants completed the Automated Reading-Span (R-span) task (Conway et al., 2005; Unsworth et al., 2005). Working memory capacity was included to partial out variance in mental math ability attributable to more general cognitive factors. In each sub-trial of the R-Span task, participants verified the semantic sensibility of a grammatically valid English sentence and were subsequently presented with a single letter. Performance on the verification task was maintained at ≥85% accuracy for all but two participants. (Because results did not differ whether we retained or excluded these participants, and because the working memory task is used only as a covariate of indirect interest, we retained these participants.) Each trial consisted of three to seven sub-trials, at the end of which participants were asked to recall the letters in the same order that they saw them. If all letters were correctly recalled for that trial in the correct order, the score for that trial was the number of letters for that trial; if any recall errors were made, the score for that trial was zero. Total scores were summed across all trials (range: 0–75), with a higher value indicating higher working memory capacity. This measure was included to control for general cognitive capacity where math ability was a variable of interest.

## Results

All analyses were performed in R v. 3.1.2 and SPSS v. 22.

To test the specificity of effects to math anxiety, trait anxiety was included as a control variable in all analyses involving math anxiety. To assess associations specific to inclusion of math in self (rather than broad inclusion of other people or concepts in self), self-friend overlap and self-literature overlap were included as control measures in all analyses involving self-math overlap. To partial out variance in general cognitive capacity, working memory capacity was included as a control measure in all analyses involving math ability. To test that effects were specific to math ability and math bias, rather than general academic ability or bias, perceived literature ability and literature bias were included as control measures in all analyses involving perceived math ability and math bias, respectively. Because females tend to have higher levels of math anxiety (Hyde et al., 1990), gender was included as a covariate in all analyses involving math anxiety. Relations between variables are presented as *r* or partial-*r* values except in the case of moderation and mediation analyses, where unstandardized betas and standard errors are presented instead for ease of interpretation.

### Validation of Self-Math Overlap

We predicted that identifying oneself strongly with math (i.e., having higher self-math overlap) would be associated with greater valuation of math. As expected, self-math overlap and valuation of math are highly positively correlated, *r*_179_ = 0.731, *p* = 2E-31. This association was unique to self-math overlap, as the partial correlation remained significant when controlling for self-friend overlap and self-literature overlap, partial-*r_177_* = 0.724, *p* = 2E-30. Self-math overlap is also positively correlated independently with each of the two PISA scales (which we combined to compute a composite measure of valuation of math). Self-math overlap is associated with greater intrinsic interest in math, *r*_179_ = 0.725, *p* = 8E-31 (controlling for covariates, partial-*r*_177_ = 0.715, *p* = 3E-29), and with greater instrumental/extrinsic interest and motivation in math, *r*_179_ = 0.516, *p* = 1E-13 (controlling for covariates, partial-*r*_177_ = 0.513, *p* = 2E-13). A significant Steiger’s *t*-test (Steiger, 1980) indicates that the association of self-math overlap with intrinsic interest in math is stronger than the association with instrumental/extrinsic interest, *p* = 3E-05 (controlling for covariates, *p* = 7E-05).

We also hypothesized that individuals who had higher self-math overlap would have lower math anxiety. Indeed, self-math overlap was inversely related to math anxiety, *r*_179_ = -0.610, *p* = 8E-20. This effect held even when controlling for self-friend overlap, self-literature overlap, trait anxiety, and gender, partial-*r_175_* = -0.567, *p* = 2E-16.

### Moderation Analyses: Self-Math Overlap, Math Performance, and Math Anxiety

Using correlational analyses, we next replicated the well-established negative relation between math performance and math anxiety, *r*_179_ = -0.355, *p* = 9E-07. This effect maintained even when controlling for trait anxiety, working memory capacity, and gender, partial-*r_176_* = -0.387, *p* = 9E-08.

To assess whether self-math overlap might moderate the negative association between math performance and math anxiety, we entered self-math overlap, math performance, and their interaction term as simultaneous predictors of math anxiety in a linear regression model. If the association between math performance and math anxiety depends on an individual’s self-math overlap, then we should see a statistically significant interaction term between self-math overlap and math performance. This is indeed what we observed: self-math overlap significantly moderated the association between math performance and math anxiety, *B* = 0.099, *SE* = 0.029, *t*(177) = 3.443, *p* = 0.001, 95% CI*_B_* = [0.042, 0.156], such that the association between math performance and math anxiety weakened with higher levels of self-math overlap. This moderation held even when controlling for self-friend overlap, self-literature overlap, trait anxiety, working memory capacity, and gender, *B* = 0.092, *SE* = 0.026, *t*(172) = 3.552, *p* = 5E-04, 95% CI*_B_* = [0.041, 0.144] (see **Table 2**). This effect cannot be attributed to differences in variability in math performance or math anxiety at different levels of self-math overlap, as the moderation is robust even in non-parametric analyses, *B* = 0.158, *SE* = 0.080, *t*(177) = 1.978, *p* = 0.050, 95% CI*_B_* = [3E-04, 0.314] (controlling for all covariates, *B* = 0.142, *SE* = 0.070, *t*(172) = 2.025, *p* = 0.044, 95% CI*_B_* = [0.004, 0.282]).

**Table 2:** Self-math overlap moderates the association between math performance and math anxiety.

	Math anxiety	
Variable	*B*	95% *CI*
Math performance	-0.468^∗∗^	[-0.659, -0.277]
Self-math overlap	-9.911^∗∗^	[-12.649, -7.173]
Self-math overlap × Math performance	0.092^∗∗^	[0.041, 0.144]
Self-friend overlap	2.204^∗^	[0.518, 3.552]
Self-literature overlap	-0.269	[-1.680, 1.142]
Trait anxiety	0.565^∗∗^	[0.360, 0.771]
Working memory	-0.023	[-0.160, 0.114]
Gender	5.325^∗^	[0.675, 9.976]
Constant	35.539^∗∗^	[18.431, 52.648]
*R^2^*	0.573	
*F*	28.87^∗∗^	

*Covariates are shown in grey. Outcome: Math Anxiety. *N* = 181. CI, confidence interval. ^∗^*p* < 0.01, ^∗∗^*p* < 0.001*.

**FIGURE 2:**
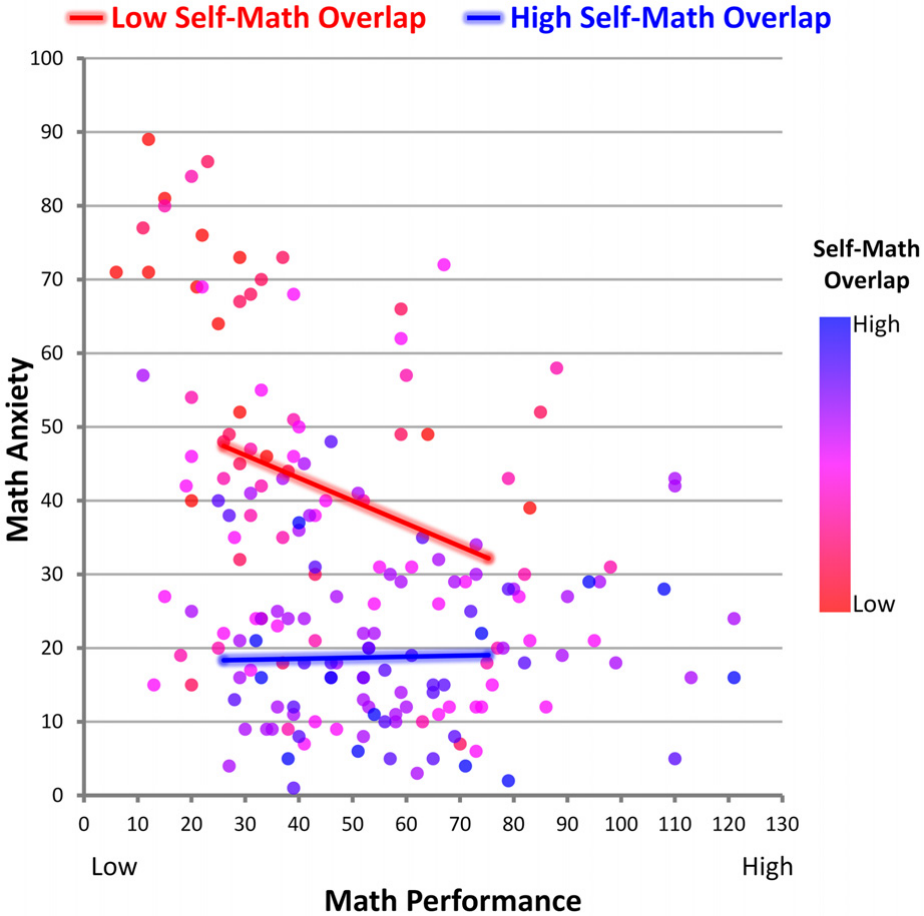
**Self-math overlap moderates the association between math performance and math anxiety**. Among individuals higher in self-math overlap, the negative association between math performance and math anxiety is ameliorated. Data points are color coded by their level of self-math overlap, where blue indicates high overlap and red indicates low overlap. Note that although redder points (lower self-math overlap) exhibit a negative association between math anxiety (*y*-axis) and math performance (*x*-axis), bluer points (higher self-math overlap) exhibit no association. This can also be seen in the overlay figure, which is a line graph based on simple slopes of the data. Among individuals with lower self-math overlap (red line; -1 *SD* in self-math overlap), those exhibiting lower math performance exhibit higher levels of math anxiety than those exhibiting higher math performance, but among individuals with high self-math overlap (blue line; +1 *SD* in self-math overlap), there is no significant association between math performance and math anxiety. Lines are drawn from -1 SD to +1 SD in math performance.

To decompose this interaction, we followed the recommendations of Aiken and West (1991) to examine simple slopes of the association between math performance and math anxiety among individuals higher (+1 *SD* above the mean of self-math overlap) and lower (-1 *SD* below the mean of self-math overlap) in self-math overlap. Among individuals with lower self-math overlap, there was a negative association between math performance and math anxiety, *B* = -0.309, *SE* = 0.071, *t*(177) = -4.351, *p* = 2E-05, 95% CI*_B_* = [-0.450, -0.169] (controlling for self-friend overlap, self-literature overlap, trait anxiety, working memory capacity, and gender, *B* = -0.319, *SE* = 0.063, *t*(172) = -5.060, *p* = 1E-06, 95% CI*_B_* = [-0.444, -0.195]), such that poorer math performance was associated with greater math anxiety. By contrast, among individuals with higher self-math overlap, there was no association between math performance and math anxiety, *B* = 0.014, *SE* = 0.067, *t*(177) = 0.212, *p* = 0.832, 95% CI*_B_* = [-0.118, 0.146] (controlling for covariates, *B* = -0.018, *SE* = 0.061, *t*(172) = -0.298, *p* = 0.766, 95% CI*_B_* = [-0.139, 0.103]) (see **Figure 2**). In sum, these results clearly indicate a moderating role for self-math overlap. With increasing levels of self-math overlap, the strength of the negative relation between math performance and math anxiety diminishes until there is essentially no significant relation between math performance and math anxiety among individuals who identify most highly with math (that is, include math in one’s self). This amelioration of the negative association between math performance and math anxiety suggests that self-math overlap may protect individuals from the threat of poor math performance, rather than exacerbating the threat. Because results favor an insulating role of higher levels of self-math overlap, we next tested the proposed mechanism for this effect – namely, that individuals with higher self-math overlap would respond defensively to the threat of poor math performance (i.e., would maintain self-enhancing biases regarding their math ability).

### Mediation Analyses: Testing the Role of Self-Enhancing Perceptions of Math Ability

Given that we observed no relationship between math performance and math anxiety in individuals who are higher in self-math overlap, we next tested the extent to which individuals’ perceptions of their math ability might explain this decoupling. We expected that individuals with higher self-math overlap would exhibit greater self-serving biases regarding their math ability. We expected that this would be particularly true among individuals with higher self-math overlap who experience threat of math failure (i.e., poor math performance).

As outlined in the Methods, we computed a measure of self-serving math bias by entering math performance (as measured by the mental arithmetic task) as a predictor into a linear regression model predicting perceived math ability. Math performance significantly predicted perceived math ability, *r_179_* = 0.373,^3^ *B* = 0.016, *SE* = 0.003, *t*(179) = 5.379, *p* = 2E-07, 95% CI*_B_* = [0.010, 0.022], but explained only a portion of the variance in perceived math ability, *R*^2^ = 0.139. The residual variance (i.e., perceived math ability residualized via removal of the influence of actual math ability) serves as our measure of math bias.

Recall that we expected individuals with higher self-math overlap to exhibit greater self-serving biases. Self-math overlap was indeed positively correlated with math bias, *r_179_* = 0.610, *p* = 8E-20. This effect held even after controlling for self-literature overlap, self-friend overlap, and literature bias, partial-*r_175_* = 0.586, *p* = 1E-17. Note that we predicted that individuals with higher self-math overlap would be most likely to demonstrate such self-serving biases specifically in the presence of a threat to the self (i.e., poor math performance). To test this, we entered self-math overlap, math performance, and their interaction term as predictors in a linear regression model predicting math bias. As expected, math performance significantly moderated the association between self-math overlap and self-serving biases, *B* = -0.004, *SE* = 0.001, *t*(177) = -2.678, *p* = 0.008, 95% CI*_B_* = [-0.006, -0.001], such that the positive association between self-math overlap and math bias was strongest when math performance was poorest. This moderation held even when controlling for self-friend overlap, self-literature overlap, working memory capacity, and literature bias, *B* = -0.004, *SE* = 0.001, *t*(173) = -2.481, *p* = 0.014, 95% CI*_B_* = [-0.006, -7E-4] (see **Table 3**). Decomposing the interaction revealed that among individuals with poorer math performance (-1 *SD*), self-math overlap more strongly predicted math bias, *B* = 0.497, *SE* = 0.045, *t*(177) = 10.987, *p* = 1E-21, 95% CI*_B_* = [0.408, 0.586], than it did among individuals with better math performance (+1 *SD*), *B* = 0.314, *SE* = 0.056, *t*(177) = 5.665, *p* = 6E-08, 95% CI*_B_* = [0.205, 0.424]. These effects held when controlling for self-friend overlap, self-literature overlap, working memory capacity, and literature bias. Among individuals with poorer math performance, self-math overlap more strongly predicted math bias, *B* = 0.495, *SE* = 0.046, *t*(173) = 10.688, *p* = 9E-21, 95% CI*_B_* = [0.403, 0.586], than it did among individuals with better math performance, *B* = 0.322, *SE* = 0.056, *t*(173) = 5.704, *p* = 5E-08, 95% CI*_B_* = [0.211, 0.434].

**Table 3:** Math ability moderates the association between self-math overlap and math bias.

	Math bias	
Variable	*B*	95% *CI*
Math performance	0.003	[-0.008, 0.013]
Self-math overlap	0.586^∗∗∗^	[0.440, 0.732]
Self-math overlap × Math performance	-0.004^∗^	[-0.006, -7E-4]
Self-friend overlap	-0.068	[-0.150, 0.015]
Self-literature overlap	0.021	[-0.061, 0.103]
Working memory	-0.003	[-0.011, 0.004]
Literature bias	-0.039	[-0.163, 0.089]
Constant	-1.002^∗^	[-1.775, -0.229]
*R^2^*	0.459	
*F*	20.92^∗∗∗^	

*Covariates are shown in grey. Outcome: math bias. *N* = 181. CI, confidence interval. ^∗^*p* < 0.05, ^∗∗∗^*p* < 0.001*.

**FIGURE 3:**
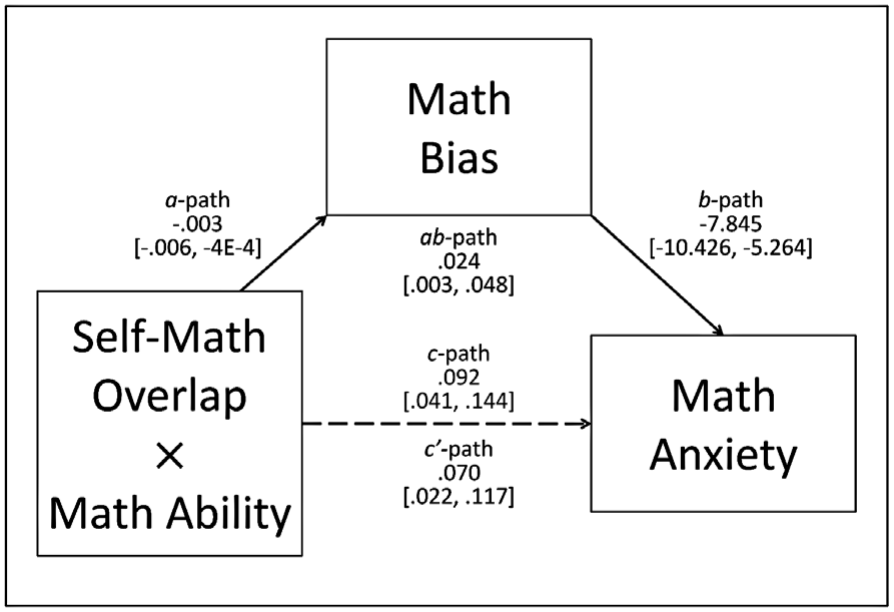
**Math bias was a significant partial-mediator of the interactive effect of self-math overlap and math ability on math anxiety, indicating that the relationship between the interaction of self-math overlap and math ability on math anxiety can be explained at least partially by levels of math bias**. In particular, having higher self-math overlap and lower math ability is associated with lower math anxiety through higher self-serving math biases. Beta-coefficients and 95% confidence intervals from a model including self-literature overlap, self-friend overlap, trait anxiety, working memory capacity, literature bias, and gender as covariates are displayed. Note that when confidence intervals do not include zero, this indicates statistical significance.

If individuals with higher self-math overlap respond defensively to threats to the self, then the decoupling of the relationship between math performance and math anxiety by self-math overlap should be explained by their self-serving biases. That is, the *moderating* effect of self-math overlap (**Table 2**) on the association between math performance and math anxiety should be *mediated* by self-serving (math) biases (i.e., mediated moderation, sometimes referred to as moderated mediation; see Hayes, 2013, pp. 357–381). In this model, the moderating (i.e., interaction) term “Math Performance × Self-Math Overlap” is in effect the predictor variable, math anxiety is the outcome variable, and math bias is the mediator (see **Figure 3**). In the preceding analyses, we demonstrated that self-math overlap and math performance interactively predict both (1) the mediator (math bias, via the *a*-path; see preceding paragraph and **Table 3**) and (2) the outcome variable (math anxiety, via the *c*-path; see preceding section and **Table 2**). Next, we assessed the association between the mediator (math bias) and the outcome variable (math anxiety; the *b*-path). Math bias was negatively associated with math anxiety, *r_179_* = -0.625, *p* = 5E-21, even when controlling for trait anxiety, literature bias, and gender, *r_176_* = -0.587, *p* = 7E-18. This provides circumstantial evidence for mediated moderation, such that the interaction of self-math overlap and math performance may exhibit a significant indirect effect on math anxiety through math bias. However, we need to directly test for the significance of the indirect effect (*ab*-path), and test what, if any, of the original (*c*) path remains after accounting for the mediator’s contribution.

We did this using the PROCESS macro v. 2.13 in SPSS (Model 8)^4^. We tested this moderated mediation model using the bootstrapping method with 1,000 iterations (Preacher et al., 2007). As predicted, the confidence interval for the indirect (i.e., mediation) effect of the “self-math overlap × math performance” interaction via math bias on math anxiety (*ab*-path) did not cross zero, *B* = 0.036, *SE* = 0.014, 95% CI*_B_* = [0.011, 0.064], indicating statistically significant mediation. The original direct effect of self-math overlap × math performance on math anxiety (*c*-path) was therefore reduced by including math bias as a mediator to *B* = 0.064, *SE* = 0.026, *t*(176) = 2.425, *p* = 0.016, 95% CI*_B_* = [0.012, 0.115] (*c’*-path). Controlling for all covariates (self-literature overlap, self-friend overlap, trait anxiety, working memory capacity, literature bias, and gender), the *ab*-path remained significant, *B* = 0.025, *SE* = 0.011, 95% CI*_B_* = [0.003, 0.048], and the *c*-path remained reduced, *B* = 0.070, *SE* = 0.024, *t*(170) = 2.907, *p* = 0.004, 95% CI*_B_* = [0.022, 0.117] (*c*’-path; **Figure 3**). Importantly, note that the direct effect of self-math overlap × math performance on math anxiety remained statistically significant even when including math bias as a mediator, indicating only partial mediation. Thus, the decoupling observed between math performance and math anxiety as a function of self-math overlap can be explained only in part by individuals’ biased perceptions of their math performance.

## Discussion

The present study indicates that the degree to which one incorporates math into one’s self, self-math overlap, may be important for understanding math anxiety. We demonstrate that higher inclusion of math in the self is associated with higher levels of math valuation and lower levels of math anxiety. In doing so, this study is to our knowledge the first to directly link research on self-expansion and the inclusion of other in self to math anxiety. Given the simplicity and visual nature of this single-item measure, we believe it may hold great promise for understanding the cognitive, social, and emotional aspects of math and math anxiety, particularly in educational contexts. For example, here we show that self-math overlap may be important for decoupling the deleterious relationship between math performance and math anxiety. Among individuals with higher levels of self-math overlap, the typically observed negative relation between math ability and math anxiety is all but eliminated.

Notably, this result helps distinguish between two competing hypotheses regarding the interplay between cognitive and affective factors in math anxiety and math performance. From one perspective, highly valuing mathematics (i.e., so much so that math becomes integrated into one’s sense of self) might make poor math performance a particularly worrying and anxiety-provoking experience. Valuing math (an affective factor) could compound an already recursive negative feedback cycle between poor math performance and math anxiety by exacerbating distractions and worries which tax additional cognitive resources. However, it instead appears that valuing mathematics so highly that one includes math in one’s sense of self in fact shields the individual from maladaptive processes which can impair math performance and provoke anxiety. Our results also indicate a mechanism by which this decoupling occurs.

Specifically, it appears that self-math overlap may protect individuals from math anxiety – at least in part – through self-serving biases. A long line of research in social psychology demonstrates that individuals are motivated to hold the self in positive regard, and the present study rests on the well-established finding that individuals feel threatened when a valued part of their self is evaluated negatively (Greenwald, 1980; Steele, 1988, 1997). When one’s ability to maintain positive self-perceptions is thwarted, individuals exhibit a number of defensive biases (Greenwald, 1980; Kunda, 1987, 1990; Dunning et al., 1995; Sherman and Cohen, 2002, 2006). Against this backdrop, our results indicate that individuals with higher self-math overlap appear to deal with the threat to self-integrity posed by the prospect of poor math performance by deluding themselves into believing their math performance is better than it actually is. In particular, we demonstrate that individuals higher in self-math overlap show relatively stronger self-enhancing biases in the math domain, and these biases explain – at least in part – the decoupling of the typically negative relation between math performance and math anxiety. Somewhat speculatively, the present study suggests that such biases may have protected these individuals from the pernicious and cognitively taxing loop between actual poor math performance and anxiety about poor math performance.

It is important to note that self-serving biases may not always be protective. Although self-serving biases are fundamental to mental health (Taylor and Brown, 1988), it is possible that such biases may lead to demotivation on future tasks (Kernis et al., 1988). To the extent to which individuals with strong self-enhancing biases about their math ability are demotivated to put forth the effort to study (perhaps believing that their effort is unnecessary for good performance given their perceived strong math abilities), self-enhancing biases may actually have the counterintuitive effect of eventually diminishing math performance. Although speculative, it is possible that individuals have higher self-math overlap simply because they recognize the societal importance of math (Parsons and Bynner, 2005; Nelson et al., 2008; Reyna et al., 2009, see also Pekrun, 2006), in which case *believing* that one is ‘good’ at math may be enough to satisfy external demands for strong math skills (c.f., Ryan and Deci, 2000) and may demotivate future effort. On the other hand, if individuals incorporate math into the self out of an inherent interest in or appreciation of math, self-enhancing biases likely increase their feelings of competence and facilitate their effortful engagement in mathematics (c.f., Ryan and Deci, 2000). Although it is likely a combination of extrinsic and intrinsic factors that lead individuals to incorporate math into their self, self-math overlap was more strongly related to intrinsic interest in mathematics than to instrumental interest in the present study. Thus, it seems more likely that self-enhancing biases about one’s math abiltiy work more in a protective fashion, preserving intrinsic interest in math, and so perhaps encouraging future interest and engagement in mathematics.

Yet, although math self-serving biases explain a decoupled relation between math performance and math anxiety as a function of self-math overlap, it is important to note that math self-serving bias only partially mediated this effect. In other words, self-serving biases explain only a portion of the variance in math anxiety as a function of self-math overlap and math performance. Therefore, additional processes to explain this relationship must be at play. This is significant because it suggests that the mechanism by which self-math overlap predicts a decoupling of math performance and math anxiety cannot merely be reduced to individuals’ self-serving biases in their perceptions of their math ability, i.e., their ‘math self-concept.’ Previous work has demonstrated that greater perceptions of one’s own math abilities are associated with lower math anxiety (Meece et al., 1990; Pajares and Miller, 1994; Ahmed et al., 2012). If our analysis had exhibited full mediation, one might conclude that our measure of self-math overlap was simply serving as a proxy for math self-concept, and did not contribute anything novel to our understanding of the psychosocial factors which relate to math anxiety. However, this was not the case, indicating that self-math overlap likely bestows additional protective advantages with respect to math anxiety. One potential protective mechanism by which self-math overlap decouples the negative rleation beween math performance and math aniety may be the extent to which those higher in self-math overlap exhibit intrinsically motivated regulatory strategies (e.g., better emotional regulation when doing math; c.f., Ryan and Deci, 2000). Future work might examine whether such regulatory strategies explain additional variance in the decoupling of the negative relation between math performance and math anxiety by self-math overlap.

It is also noteworthy that individuals with lower levels of self-math overlap continued to exhibit a strong negative relation between math performance and math anxiety in the present study. This is somewhat counterintuitive given that individuals lower in self-math overlap value math less than those who have higher self-math overlap. One might have predicted an ameliorated or decoupled relation between math performance and math anxiety at relatively low levels of self-math overlap because if individuals do not value math as part of their identity, they can hardly be expected to feel pressure to perform well in math or to be anxious about their performance. However, this is not what we observe in the present study. Rather, the finding that lower self-math overlap individuals have a *stronger* negative relation between math performance and math anxiety suggests that they are potentially even more susceptible to worried rumination or distraction that exacerbates the negative loop between math anxiety and math performance. In other words, this result undercuts the notion that those who ‘care’ less about math are not math anxious or are immune to the pernicious relation between math anxiety and math performance. Although our results show this can be partly attributed to less positively biased perceptions of math ability among those with lower self-math overlap, the mediation effect was only partial. In other words, other more extrinsic factors (e.g., awareness of the importance of math skills for socially desirable outcomes) might be at work in these individuals.

On a methodological note, when discussing a decoupling of a negative relation between math performance and math anxiety through self-enhancing biased assessments of one’s math performance, it is worth considering how these constructs are measured and the assumptions therein. Here, we measured performance via a difficult mental arithmetic task. However, more advanced mathematics such as algebra, geometry, calculus, etc., may – at least in the minds of the students assessed here – have relatively little to do with arithmetic skill. Though math tends to be a cumulative discipline, and considerable research has linked basic arithmetic and numerical skills with more advanced math abilities (e.g., Blaylock and Kopf, 2012; Lourenco et al., 2012; Price et al., 2013), individuals’ *perceptions* of their math ability may not be so closely related to their arithmetic and numerical skills, especially if they have extensive training in advanced mathematics. Thus, the way in which math performance and perceptions of math ability are operationalized requires particular consideration, especially when studying those who are especially advanced in mathematics relative to more typical populations. Future work might consider whether self-math overlap exerts a uniform effect on the relation between math performance and math anxiety across more advanced mathematical contexts

Additionally, our measure of perceived math ability (from which we computed a measure of self-enhancing math bias) was a single item measure, and thus may potentially elicit objections that it fails to capture nuances in individuals’ assessments of their math abilities. However, the correlation between perceived and actual math ability in the present study (*r* = 0.37), was in fact slightly *above* the overall average of such correlations (*r* = 0.33) observed across cognitive domains in the recent meta-analysis by Freund and Kasten (2012). Moreover, looking just at the relation between perceived and actual performance in the numerical domain with multi-item measures of perceived ability, the typical correlation was 0.40, which is only slightly higher than the relation observed here. Thus, although future work will no doubt further elucidate the nuanced relation between perceived and actual math ability, our measurement of these variables here does not seem to have unduly compromised our results.

On a practical note, our measure of self-math overlap is both brief and, given its visual nature (**Figure 1**), quite easy to understand. Many previous measures that examine a potentially related construct, math identification, have relied heavily upon positive perceptions of math abilities. Although we have shown that self-math overlap is positively related to perceived math ability, it is far from a one-to-one correlation. In fact, one of our critical results is that math self-serving bias only partially mediates the interactive effect of self-math overlap and math performance on math anxiety. This suggests that when it comes to math anxiety (and in particular the relation between math anxiety and math performance), self-math overlap appears to bring additional explanatory power. Moreover, the simplicity of this measure may also be well-suited to testing in a range of environments and populations, including cross-cultural and developmental contexts. Such work is particularly important given that the sample for the present study was comprised of ‘WEIRD’ undergraduate subjects (that is, subjects from Western, educated, industrialized, rich, and democratic backgrounds; Sears, 1986; Henrich et al., 2010) and may not generalize to other populations. Finally, as the results here suggest, self-math overlap may also prove useful for reducing the pernicious relation between math performance and math anxiety, and vice versa. To the extent that it is possible to explicitly intervene in ways that increase incorporation of math into one’s sense of self, for example, such interventions may help reduce, delay, or even prevent some of the deleterious effects of math anxiety on math education.

In sum, the current work demonstrates that including math in one’s sense of self – self-math overlap – predicts reduced math anxiety and a decoupling of the association between math ability and math anxiety. Individuals with higher self-math overlap exhibit biased perceptions of their own math ability, especially to the extent that they suffer from threat of poor math performance. These biases in turn partially explain the decoupling of the link between math performance and math anxiety in these individuals, though unique variance remained attributed to self-math overlap, suggesting a still deeper connection between this novel construct and math anxiety that warrants further investigation. Moreover, even though those lower in self-math overlap tended to value math less, we nevertheless observed a stronger negative relation between math anxiety and math performance in these individuals. This work thus presents a promising avenue for understanding the nuanced relation between math ability and math anxiety, and it provides a clear theoretical link between ongoing research in math anxiety and social psychological research on the benefits of including others in one’s sense of self. Studying the strategies that individuals with high self-math overlap utilize in anticipation and performance of mathematics may inform methods for effectively intervening and disrupting the downward spiral between poor math performance and math anxiety.

## Conflict of Interest Statement

The authors declare that the research was conducted in the absence of any commercial or financial relationships that could be construed as a potential conflict of interest.

## Footnotes

1 Typical items include “I have always done well in math” and “I am good at math” (Spencer et al., 1999; Smith and White, 2001); for a recent and interesting exception, see Cribbs et al. (2015).

2 Throughout the manuscript, we refer to the ‘threat that poor math performance poses to one’s sense of self-integrity (to the extent that math is important to one’s sense of self, as measured here by self-math overlap)’ more parsimoniously as ‘threat of poor math performance,’ ‘threat of math failure,’ ‘threat to self-integrity,’ or ‘threat to the self.’ Note, however, that this is not meant to imply an experimental manipulation of threat.

3 Note that this correlation is typical of the association between self-assessments of cognitive abilities and objective measures of cognitive abilities, where the average such correlation was found to be *r* = 0.33 in a recent meta-analysis (Freund and Kasten, 2012).

4 PROCESS Model 8 was run using the following variable assignments: *X* = math performance, *W* = self-math overlap, *M* = math bias, *Y* = math anxiety. Covariates were applied in both the moderated and mediated components of the model.
